# Supplementary material for: Protocol for 3D digital dynamic histomorphometry of mouse bone via time-lapse registration of serial microCT scans
Source: STAR Protoc. 2026 Aug 25;7(3):104787. doi: 10.1016/j.xpro.2026.104787 (PMC13543904; doi:10.1016/j.xpro.2026.104787)
Supplement: Supplementary file 1 [file mmc1.pdf]

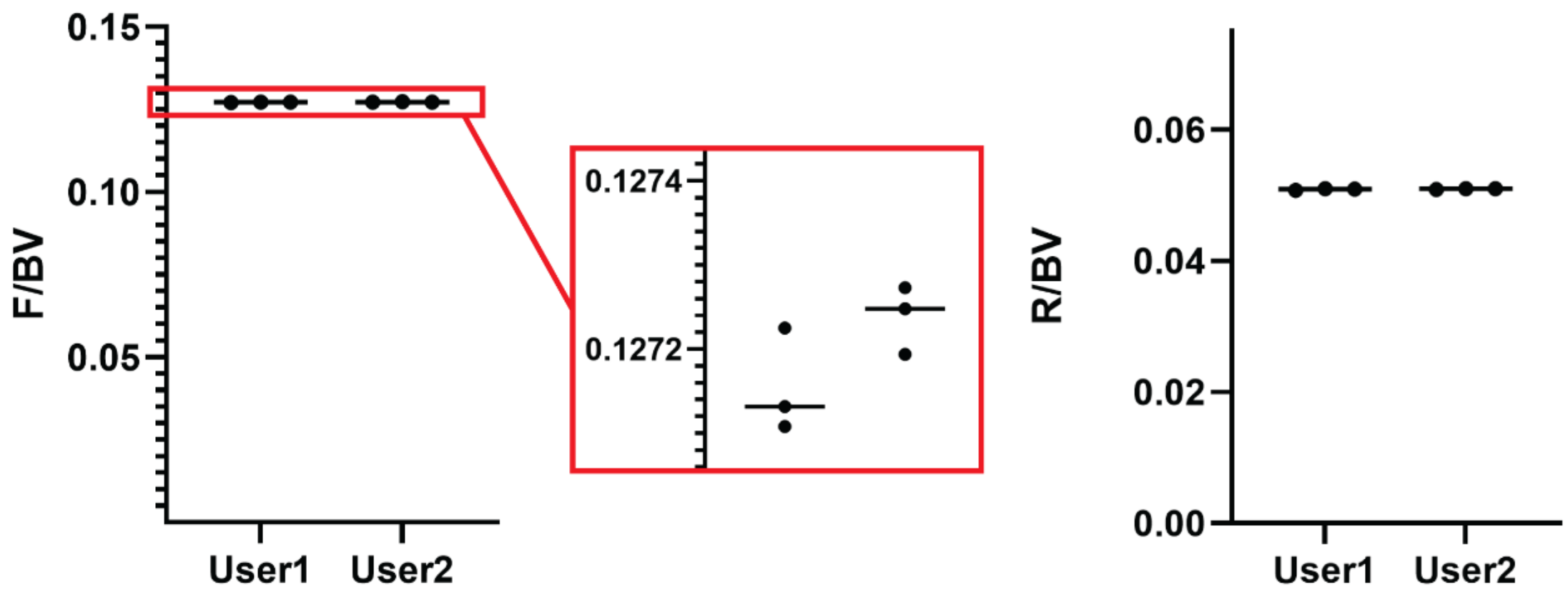

Supplemental figure S1: Reproducibility intra and inter operators (Related to Expected Outcomes). Two independent operators registered the same mouse tibia midshaft dataset three times and quantified the formation (F) and resorption (R) volume normalized by the PreScan bone volume (BV). F/BV and R/BV are dimensionless ratios representing the volume of newly formed or resorbed bone normalized to the total bone volume within the analyzed region.
